# Incidence and complications of orthopaedic screw protrusion

**DOI:** 10.1530/EOR-2024-0147

**Published:** 2025-06-30

**Authors:** Rasi Mizori, Mueed Ijaz, Mohamed Hashem, Ruben Doyle, Yasser Al Omran, Omar Musbahi

**Affiliations:** ^1^ King’s College London Guy’s Campus, London, UK; ^2^ Frimley Park Hospital NHS Foundation, Frimley, Surrey, UK; ^3^ Imperial College London, London, UK; ^4^ Royal Free Hospital, London, UK

**Keywords:** Orthopaedic surgery, screw protrusion, neurovascular injury, avascular necrosis, pseudoarthrosis, tendonitis/tendon rupture, delayed union, orthopaedic surgery screw complications

## Abstract

**Purpose:**

- This scoping review examines the epidemiology and incidence of complications associated with incorrect screw length in orthopaedic trauma surgeries, aiming to underscore its impact on clinical outcomes and healthcare costs.

**Methods:**

- Guided by Arksey and O'Malley’s framework and PRISMA guidelines, a thorough search was conducted across PubMed, Web of Science, Cochrane, and Google Scholar up to April 2024. Studies included must be human-based orthopaedic procedures involving screw length, published in peer-reviewed journals with full text available, and report complications from incorrect screw length; studies excluded were systematic reviews/meta-analyses. Paper characteristics and data on incident rates of complications due to screw protrusion were extracted and presented in a supplementary table using Microsoft Excel.

**Results:**

- Of 2,285 studies identified, 31 met the inclusion criteria. Incorrect screw length led to severe complications, including neurovascular damage (7 studies), avascular necrosis (4 studies), delayed union or pseudoarthrosis (9), tendinitis/tendon rupture (12 studies), and pain (10 studies). Notably, improperly sized screws increased neurovascular and avascular necrosis complications, especially in weight-bearing joints. In addition, incorrect screw length was linked to higher hardware removal rates, contributing to increased healthcare costs. Limitations include the inability of some studies to definitively attribute complications to screw protrusion and the exclusion of cases involving joint collapse or solely mechanical complications.

**Conclusion:**

- This review underscores the importance of precise screw length selection to prevent complications and improve surgical outcomes. It calls for further research on the medico-legal and economic impacts of screw length errors and the need for better surgical practices.

## Introduction & background

Orthopaedic surgery is a fundamental aspect of restoring function and alleviating pain in patients with musculoskeletal injuries. These conditions significantly impact public health, representing the most substantial disease burden in developed countries (1, 2). In the United States alone, about 2 million fracture-fixation devices are inserted annually (3). This statistic is consistent with the 2017 Global Burden of Disease Study findings, which identified musculoskeletal conditions as the leading contributors to global disability (4).

Furthermore, the incidence of fractures has been increasing due to the worldwide ageing population, with 178 million new cases recorded in 2019, a 33.4% increase compared to 1990 (4). This trend combined with the predicted growth of the global population aged 65 and above from 10% in 2022 to 16% in 2050, highlights a need to address this growing epidemic and ensure hospitals and surgeons are well equipped to deal with this situation.

The safety and efficacy of medical devices in orthopaedic trauma are crucial to patient outcomes during surgery. These devices, which range from implants such as intramedullary nails and trauma plates to power tools and surgical assistive aids, vary significantly in efficacy and cost (5). Among these devices, screws are the most commonly used implant. The bone screw system market size was valued at around USD 1.7 billion in 2023, and over 1 billion orthopaedic trauma screws are inserted by surgeons annually (6). This highlights their critical function in various T&O procedures, especially in the attachment of implants to bone, bone/bone fixation, and bone/soft tissue fixation (7).

Currently, manual depth gauge (MDG) is the gold standard method to estimate screw length (8). MDGs are used to gauge the depth of the holes drilled in bones, ensuring the correct screw length is selected and inserted. Despite being the gold standard, there is a risk of selecting inappropriate screw lengths. Excessively long screws can cause numerous complications, including nerve injury, pseudoarthrosis, avascular necrosis, tendinitis, and more, significantly impacting patient outcomes. Studies indicate that tendinitis and tendon rupture can occur in 65% of cases associated with excessively long screws (9). As well as the associated medical complications, there are significant medicolegal and economic implications that are important to consider. Therefore, given that the screw market size is projected to reach USD 2.8 billion by 2032 (4), it has also become increasingly important to consider both medical and cost complications.

Despite the vast number of screws being inserted in T&O surgeries annually worldwide, the literature gap on the epidemiology and incidence of screw protrusion and screw-related complications is stark. This scoping review aims to spotlight the critical issue of excessively long screws being used in orthopaedic surgeries, delving into the complications they breed, the additional healthcare costs they incur, and the urgent need for improved practices and/or technologies to mitigate this problem.

## Methods

### Study design

This scoping review was undertaken in line with Arksey and O’Malley’s methods. Search and screening results are presented according to ‘The Preferred Reporting Items for Systematic Reviews and Meta-Analyses’ (PRISMA) guidelines. The review aimed to identify the prevalence of excessively long screw usage in surgeries and the associated patient complications and costs associated with MDG. This review was not registered in a formal review database, as it was conducted following scoping review guidelines rather than a systematic review framework. No amendments were made to the methodology during the course of this review, as the review was planned and executed in alignment with the predefined scoping framework.

### Search strategy

A literature search was performed using four online databases: PubMed, Web of Science, Cochrane, and Google Scholar in April 2024. They were restricted to full-text articles written in English using the following keywords: complications, adverse events, adverse outcomes, poor outcomes, injury, surgery, orthopaedic surgery, screw length, and screw depth. The search was made using Boolean search operators: ((‘complication’ or ‘poor outcome’ or ‘adverse outcomes’ or ‘adverse results’ or ‘adverse effects’ or ‘surgical complications’) and (‘screw length’ or ‘screw size’ or ‘screw depth’ or ‘depth measurement’ or ‘depth gauge’ or ‘depth control’ or ‘depth assessment’) and (‘orthopaedic’ or ‘orthopedic’ or ‘surgery’ or ‘surgeries’ or ‘surgeons’ or ‘operation’ or ‘orthopaedic procedures’)). Identified publications were imported into Rayyan. Titles and abstracts (stage 1) and full-text trials (stage 2) were independently screened by two reviewers (M I and R M). The third and fourth reviewers (Y A O and O M) were consulted where consensus could not be obtained by discussion. Two reviewers (M I and R M) extracted data independently using a standardised form. A third reviewer (L S C-M) independently checked the data for consistency and accuracy.

### Inclusion criteria

Screw length.
Orthopaedic procedure.
Human-based study.
Peer-reviewed.
Must have complications of incorrect screw length.
Full text available.

### Exclusion criteria

Systematic reviews/meta-analyses.

## Results

### Study selection

**Figure 1:**
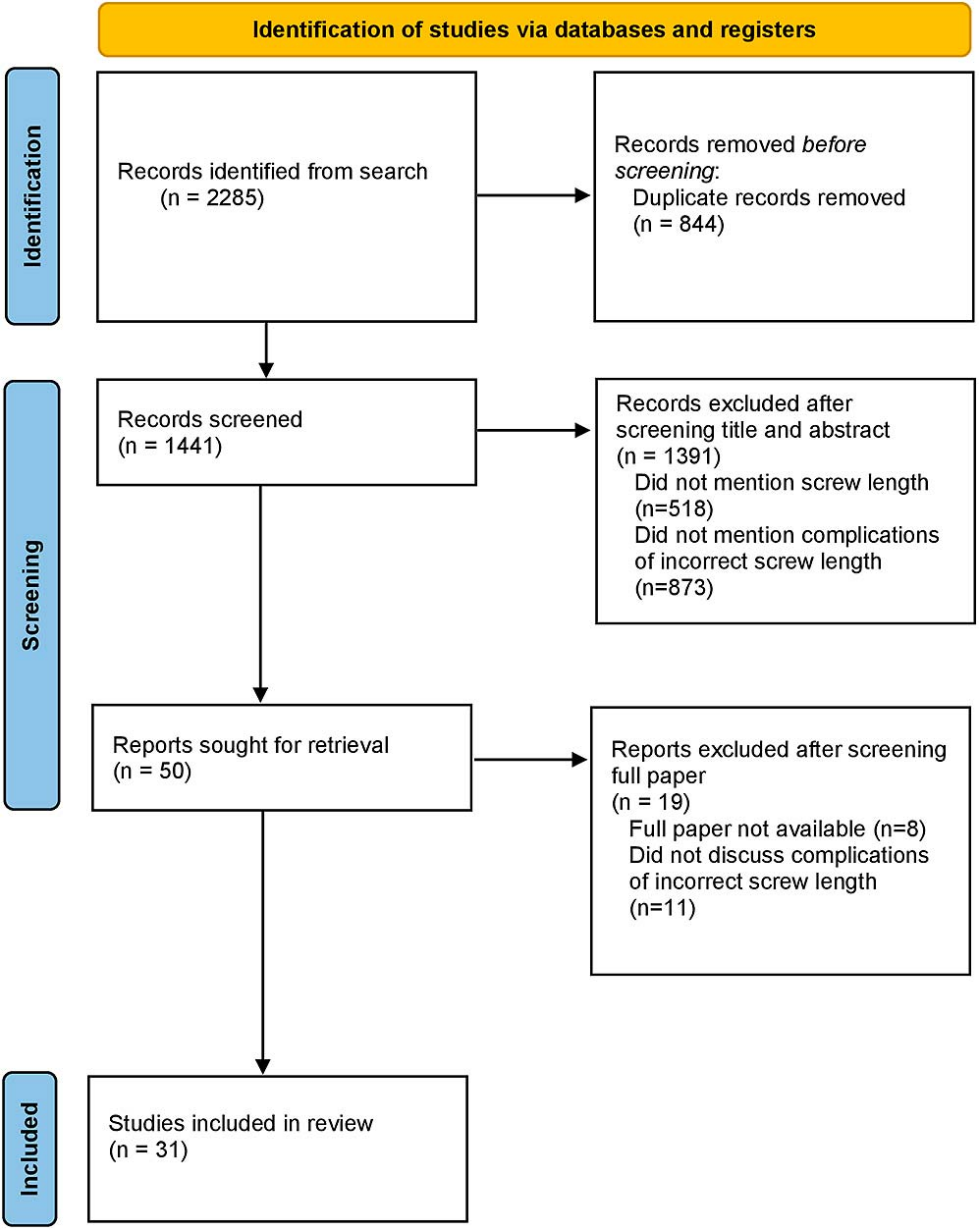
PRISMA flow diagram demonstrating the process for inclusion of selected papers in the review.

The search produced a total of 2,285 results from three databases. After removing duplicates, 1,441 articles were retrieved for review. Following a review of the title and abstract, 1,391 were removed, leaving 50 papers. In total, 31 studies that met the inclusion and exclusion criteria were included in the review (Fig. 1).

The review sought data on patient outcomes related to screw protrusion, including the incidence and types of complications (e.g. neurovascular damage, avascular necrosis, pseudoarthrosis, tendon injuries, and pain), as well as their rates and relevant follow-up periods. Other variables collected included study design, country of origin, surgery type, sample size, and follow-up duration. Assumptions about unclear or missing data were resolved through consensus discussions. Effect measures for the outcomes included incidence rates and percentage rate of complications.

The synthesis process involved selecting studies based on adherence to inclusion criteria, such as human-based studies reporting complications due to incorrect screw length, and grouping them by specific outcomes. Results were systematically presented using a data extraction table and PRISMA flow diagram. As a meta-analysis was not conducted, results were synthesised narratively, focusing on trends and commonalities across studies without applying statistical models or measures of heterogeneity. Potential causes of heterogeneity, such as differences in study design, sample sizes, and surgical techniques, were qualitatively explored but not formally analysed. Due to the descriptive nature of the synthesis, sensitivity analyses were not conducted.

### Complications of incorrect screw length

#### Neurovascular complications

Within the scope of this review, seven out of thirty-one (19%) studies reported neurovascular complications as a result of screw protrusion (10, 11, 12, 13, 14, 15, 16). Suk *et al.* described a 1.5% malposition rate in thoracic pedicle screw insertions for spinal deformities, with neurological complications in 0.8% of patients, including a transient paraparesis that resolved 3 weeks post-removal of the offending screw (10). Hamill *et al.* noted direct nerve root irritation leading to radiculopathy in their retrospective cohort study, with two cases necessitating surgical revisions to relieve symptoms and correct neurological deficits (11). A case report by Starnoni *et al.* provided a detailed account of a 36-year-old patient who experienced numbness and burning pain in the median nerve distribution after undergoing percutaneous scaphoid fracture fixation (12). A CT scan revealed complete screw displacement from the scaphoid, and its removal resulted in immediate improvement and complete resolution of symptoms 2 months postoperatively. Esses *et al.* found that incorrectly sized pedicle screw fixation of the lumbar spine led to transient neuropraxia in 2.4% and permanent nerve root injury in 2.3% of cases, with twelve of the fourteen cases with permanent injury requiring reoperation. Hu *et al.* investigated the accuracy and complications associated with the freehand C-1 lateral mass screw fixation technique, and of 196 patients, two patients (1%) complained of post-operative occipital neuralgia (14). While this was transient in one patient and resolved 2 months post-surgery, the remaining patient developed persistent neuralgia that remained at a 2-year follow-up check, ultimately leading them to be referred to the clinical pain service. Madawi *et al.* reported significant complications in their study of C1-2 trans-articular screw fixation for spinal instability, including vertebral artery lacerations and significant venous bleeding in five patients, one of whom developed progressive brainstem strokes resulting from screw-induced occlusion of the left vertebral artery (15). Lonstein *et al.* found similar results in their study as they conducted a retrospective review of 4,790 screw insertions, finding that 134 (2.8%) caused nerve injury by perforating the anterior cortex (16). Although screw removal resolved neurological issues in seven out of eight patients, marked residual weakness remained.

#### Avascular necrosis

Four out of thirty-one studies (13%) noted avascular necrosis (AVN) as a complication due to protruding screws (17, 18, 19, 20). Yang *et al.* reported a 3.1% incidence of AVN in patients treated with a locking proximal humerus plate, with an overall complication rate of 35.9% (17). Similarly, Kavuri *et al.* observed a 4.6% avascular necrosis rate in proximal humerus fractures managed with locking plates related to protruding screws (18). Wang *et al.* performed a retrospective analysis of 153 paediatric cases with femoral neck fractures, revealing that screws of larger size were associated with an increased incidence of AVN in children with femoral neck fractures treated surgically (19). Finally, Beeres *et al.* found that among patients undergoing locking plate fixation of proximal humerus fractures, 45% experienced complications, with 10% of these cases developing avascular necrosis (20).

#### Delayed union/pseudarthrosis

Seven of thirty-one papers (19%) in the search explored the impact of screw length on the rate of delayed union/pseudoarthrosis (16, 18, 20, 21, 22, 23, 24). Lee *et al.* investigated the impact of screw length, measured as vertebral body percentage (VB%), on fusion success in 85 patients undergoing anterior cervical discectomy and fusion (ACDF), finding that a VB% less than 75% significantly reduced 1-year fusion success rates from 92.9 to 64.3%, with a revision rate of 4.7% (21). Chanbour *et al.*, in a cohort study involving patients undergoing ACDF, confirmed a higher incidence of radiographic pseudoarthrosis when screws had a VB% below 75% (39.6 vs 22.1%, *P* < 0.001) (22).

Similarly, Kavuri *et al.* noted a 1.5% non-union rate in a 12-month follow-up period in proximal humerus fractures treated with locking plates (18). Lonstein *et al.*, in their study of 875 patients, observed pseudoarthrosis in 4.7% of patients using pedicle screws, correlating with 23% of the screws in 24.3% of the procedures (16). Muller *et al.* investigated the results of anterior screw fixation of odontoid fractures in twenty-eight patients and found that incorrect sizing of the screws in the odontoid, with penetration of the postero-lateral cortex, occurred in three patients (10.7%), with one patient (3.6%) experiencing non-union with persistent instability necessitating secondary posterior C1/2 fusion (23). Beeres *et al.* performed a multi-centre international study involving 282 patients and documented a 2% non-union rate among patients following locking plate fixation of proximal humerus fractures (20). Furthermore, the SWIFFT trial found that screw penetration in scaphoid fracture surgeries resulted in non-union or incomplete union of the fracture in 4% of the surgical group participants, indicating a significant risk of delayed union or pseudoarthrosis associated with screw protrusion (24).

#### Tendinitis/tendon rupture

Among the thirty-one papers included in the review, eleven (35%) identified tendinitis or tendon rupture as complications arising from incorrect screw length in surgical procedures (9, 25, 26, 27, 28, 29, 30, 31, 32, 33, 34). Three of these studies investigated distal radius fractures, with documented tendinitis rates as follows: Oc *et al.* (14.9%), Rellan *et al.* (0.9%), and Alic *et al.* (51.7%) (9, 25, 26). The remaining studies, including those by Bergsma *et al.*, Benson *et al.*, Sugun *et al.*, Bianchi *et al.*, Gyuricza *et al.*, Yamazaki *et al.* and Drobetz *et al.*, reported both tendinitis and tendon ruptures (27, 28, 29, 30, 31, 32, 33) (Supplementary Table 1 (see section on Supplementary materials given at the end of the article)). In addition, a case reported by Mayne *et al.* detailed a patient experiencing pain and discomfort post total hip arthroplasty, diagnosed with iliopsoas tendinitis. The severity of symptoms significantly decreased following the surgical removal of the screw (34).

#### Pain

Among the thirty-six studies, ten (28%) reported pain as a complication from protruding screws in orthopaedic surgeries (13, 20, 21, 24, 31, 35, 36, 37, 38, 39). Ahmed *et al.* found a 16% screw removal rate in patients with scaphoid fractures due to screw protrusion, with two patients experiencing persistent pain necessitating screw removal (35). In the SWIFFT trials, Dias *et al.* used the patient-reported wrist evaluation (PRWE) form to document pain levels after scaphoid injury (24). They found that among the patients who experienced screw penetration (65%), as assessed by CT scans at the 52-week follow-up, those with a screw penetration of 1 mm or more reported a higher PRWE score (10.8), indicative of greater pain, compared to those with screw penetration of less than 1 mm (8.9). Further, Gyuricza *et al.* noted that eleven out of twenty-eight patients required hardware removal due to pain after distal radius fractures (31). Additional studies by Esses *et al.*, Beeres *et al.*, Drosos *et al.*, and Zhu *et al.* similarly reported pain alleviation upon the removal of protruding screws (13, 20, 36, 38). Detailed data across these cases are available in Supplementary Table 1.

## Discussion

This review investigated the epidemiology and incidence of complications due to excessive screw length in orthopaedic surgeries. The review identified four main complications consistently associated with screw protrusion: neurovascular complications, avascular necrosis, delayed union or non-union, and tendinitis. Among the thirty-one papers included in this review, twenty-eight studies (90.3%) identified screw protrusion as a primary cause of complications. These findings consistently demonstrate the direct impact of screw length and positioning on patient outcomes. Three studies (9.7%), however, were unable to establish a definitive causative relationship between screw length and complications (discussed in more detail in the limitations section).

### Neurovascular complications

Due to the anatomical proximity of bone to neurovascular structures, screw protrusion can cause neurovascular complications by directly compressing or penetrating nearby nerves and blood vessels, with mechanical interference resulting in nerve irritation, neuropraxia, or permanent nerve damage (22). The cause of neurovascular compromise was most often confirmed either through imaging, as in Hamill *et al.* (11), or the resolution of symptoms after the removal of the screw, as in Starnoni *et al.* (12).

Findings from the seven studies highlight the severe risks of neurovascular complications as a result of incorrect screw length in orthopaedic surgeries. Notably, the study by Madawi *et al.* reported cases of brainstem strokes following vertebral artery laceration and venous bleeding, emphasising the critical importance of precise screw placement (15). Although many neurological issues were resolved following the removal of improperly placed screws, the persistence of residual weakness in some patients serves as a reference for the potential for irreversible damage. These findings demonstrate the critical need for meticulous pre-surgical planning, accurate surgical execution, and prompt post-surgical monitoring, as delayed recognition can result in long-term neurological deficits, loss of function, and even mortality (29).

### Avascular necrosis

Screw protrusion was found to cause AVN by disrupting the blood supply to subchondral bone through direct vascular injury or excessive mechanical compression. For instance, studies such as Wang *et al.* and Beeres *et al.* demonstrated that improperly placed or oversized screws compromised critical blood flow, particularly in weight-bearing joints, leading to ischaemic bone death (19, 20). This direct relationship between protruding screws and AVN was observed in multiple studies within the review, and the findings underscore the risk that incorrect screw length can have on maintaining the integrity of the subchondral bone supply. This is of particular concern when considering surgeries that involve weight-bearing joints, such as the hip, where the prognosis is poor (40). This review aligns with the broader literature, with studies such as the one by Lespasio *et al.* highlighting the multifactorial aetiology of avascular necrosis, with surgical intervention being an important one to consider (41).

### Delayed union/non-union

Non-union occurs when a fracture fails to heal within 9 months, with risk factors such as advanced age, comorbidities, and lifestyle habits (33, 34). The primary barrier to healing is disrupted blood supply, leading to significant health consequences (16). In addition to physiological alterations, an unfavourable biomechanical environment, such as that caused by incorrect screw length, can also significantly hinder fracture healing and contribute to pseudoarthrosis.

A review of the seven studies highlights the key role of screw length in achieving bone fusion, particularly in spinal surgeries. Lee *et al.* utilised vertebral body percentage (VB%) as a metric and demonstrated the critical threshold below which the likelihood of achieving successful spinal fusion significantly diminishes (21). This correlation was consistently observed across other studies, including the study by Chanbour *et al.* (22), who reported higher rates of radiographic pseudoarthrosis below this VB% threshold. Such findings emphasise the necessity of mechanical stability for healing and suggest that precise surgical techniques can markedly enhance patient outcomes.

### Tendinitis/tendon injury

The findings from the ten studies offer compelling evidence of the biomechanical repercussions of surgical inaccuracies, particularly the incorrect selection of screw length. As demonstrated in Supplementary Table 1, screw protrusion frequently leads to tendinitis and tendon injuries, highlighting the critical need for precise screw length determination and post-operative vigilance, especially in those with known screw protrusions.

Screw protrusion was found to cause tendinitis and tendon rupture by mechanically irritating and abrading adjacent tendons, leading to inflammation, micro-tears, and eventual rupture. This relationship is well documented in studies such as Alic *et al.*, where there was a statistically significant correlation between the protruding screw length of >1.6 mm and the presence of tendinitis (*P* < 0.05) (9). This precise data underscores the potential for policy improvements in surgical practices to minimise the risk of screw protrusion and associated complications.

### Pain

The presence of pain associated with protruding screws in orthopaedic surgeries, as highlighted in the ten studies included in this review, underscores the risk of complications in postoperative outcomes. Protruding screws can induce pain by mechanical irritation of surrounding tissues or by direct impingement on neurovascular structures. For instance, Ahmed *et al.* reported a notable rate of screw removal due to pain in patients with scaphoid fractures, indicative of the mechanical challenges associated with optimal screw placement in smaller bones (35). The persistence of pain until screw removal, as observed in multiple case studies from diverse geographical locations (37, 38, 39), further exemplifies the universal clinical significance of this issue. These case studies collectively highlight that protruding screws not only compromise structural stability but also impact patient quality of life. The immediate relief from pain following the removal of offending screws in these varied cases underscores the direct relationship between screw protrusion and pain. This observation suggests pain can be a reliable clinical indicator of potential complications related to hardware misalignment or protrusion. Consequently, early intervention to correct these hardware issues is crucial to prevent long-term sequelae such as chronic pain or even more severe complications such as tissue necrosis or infection. The studies reviewed also demonstrate a broader implication for surgical practice: the necessity for meticulous preoperative planning and intraoperative imaging to ensure accurate screw placement. The consistent findings across different studies and anatomical sites suggest that the principles of hardware placement transcend specific surgical techniques and are applicable universally across orthopaedic procedures.

### Medico-legal and economic considerations

The medico-legal and economic considerations of orthopaedic surgeries involving screw placement are profound, influencing both patient outcomes and the financial stability of healthcare institutions.

The frequency of misplacement of pedicle and lateral mass screws in spine surgery compounds the economic challenges faced by healthcare systems. Research indicates that screws are in as many as 65% of cases (24), with associated injury rates reaching as high as 23%. With nearly a third of judgments/settlements favouring the plaintiff, the financial toll is substantial, with average payouts exceeding $1.2 million per claim. These figures paint a stark picture of the financial liabilities faced by healthcare institutions, emphasising the urgent need for proactive measures to mitigate risks and minimise associated costs. Beyond individual malpractice claims, the broader economic impact is considerable. The total financial burden of procedural errors leading to combined payouts in neurosurgery claims, as reported by Sankey *et al.*, amounted to $124,943,933 over 9 years (42). These statistics underscore the pervasive nature of the issue and highlight the need for systemic interventions to improve surgical outcomes and reduce financial liabilities.

While the study by Sankey *et al.* provides valuable insights into the medicolegal and economic implications of incorrect screw length or placement in spine surgery, it is essential to acknowledge the limitations inherent in retrospective analyses and legal databases. However, despite these limitations, the findings underscore the urgent need for proactive measures to mitigate risks, improve patient safety, and reduce the financial burden of malpractice claims.

### Implications for practice and key considerations

This review highlights significant complications arising from inappropriate screw length in orthopaedic surgeries, underscoring an urgent need to refine surgical practices to improve patient outcomes. With an uptrend in fracture incidences, an ageing population, and more frequent fixation surgeries, precise screw length is paramount to avoid adverse outcomes. Innovations in surgical technology offer promising solutions to these challenges.

### Advancements in surgical techniques and tools

Technological innovations in orthopaedic surgery have focused on reducing variability and reliance on subjective surgeon judgement during procedures. Digital automated devices, such as advanced depth measurement systems and smart surgical tools, offer consistent and objective measurements, minimising the risk of human error in screw length selection (43). These technologies standardise processes by providing precise, real-time feedback and reducing inter- and intra-surgeon variability. By eliminating much of the guesswork and variability inherent in manual techniques, these advancements have the potential to enhance patient safety, reduce complication rates, and contribute to more predictable surgical outcomes.

## Limitations

While the majority (90.3%) of included studies directly attributed complications to screw protrusion, three studies could not establish a definitive causative relationship. In these studies, complications were attributed to broader biomechanical or surgical factors rather than screw protrusion alone. Gyuricza *et al.* reported complications such as tenosynovitis, tendon rupture, and prominent screws, but noted other factors such as infection (31). Similarly, Lonstein *et al.* identified pseudoarthrosis and nerve root irritation but attributed these to broader biomechanical issues (16). Dias *et al.* also highlighted implant-related complications without definitively attributing these complications to screw length (24).

Another key limitation of our review revolves around the common practice of discussing clinical findings in morbidity and mortality meetings between local units without subsequent publication. This often results in significant clinical insights that remain under-reported in the scientific literature. The absence of such data can skew the apparent prevalence and implications of the issue being studied, making it an important limitation for us to note in our review. In addition, our review focused exclusively on complications arising from screw protrusion due to excessive screw length, excluding cases where protrusion resulted from joint collapse. We also did not isolate studies explicitly identifying articular protrusion as a mechanical complication. Our inclusion and exclusion criteria prioritised studies linking articular protrusion to patient-centred clinical outcomes, potentially overlooking mechanical-only perspectives. Future research should aim to integrate both the mechanical and clinical dimensions of articular protrusion to provide a more comprehensive understanding of their interconnection and impact.

## Conclusion

This review shows the significant implications of screw protrusion in orthopaedic trauma. Thirty-one studies were included, and complications ranged from avascular necrosis (4.6%) to tendinitis (51%). These complications were associated with a high reoperation rate. Further studies are required to understand the health economic costs and the extent of medicolegal complications.

## Supplementary materials



## ICMJE Statement of Interest

The authors declare that there is no conflict of interest that could be perceived as prejudicing the impartiality of the work reported.

## Funding Statement

This research was supported by the AO Trauma Research Grant and the St Bartholomew’s Medical College Contingency Grant.

## Data Availability

All data used in this review, including the extracted data and template forms, are available upon reasonable request from the corresponding author.
