# Supplementary material for: Incidence and complications of orthopaedic screw protrusion
Source: EFORT Open Rev. 2025 Jun 30;10(7):562–9. doi: 10.1530/EOR-2024-0147 (PMC12229280; doi:10.1530/EOR-2024-0147)
Supplement: Supplementary file 1 [file Supplementary_materials.pdf]

# Incidence and Complications of Orthopaedic Screw Protrusion

## Appendices

### APPENDIX 1

*Supplementary Table of Included Studies*

| | Author | Study YEAR | Study COUNTRY | Study TYPE | Surgery type/screw insertion area | NUMBER OF PATIENTS IN STUDY | NUMBER OF PATIENTS WITH A SCREW PROTRUSION (%) | NUMBER OF PATIENTS WITH A COMPLICATION (%) | FOLLOW UP PERIOD (MONTHS) | |
|---|---|---|---|---|---|---|---|---|---|---|
| 1 | Bergsma et al (1). | 2018 | Australia | Prospective cohort study | Distal radius fractures | 100 | 26 (26%) | 1 (1%) | 12 | Tendon rupture/tendonitis |
| 2 | Benson et al (2). | 2006 | United States | Case series | Distal radius fractures | 2 | 2 (100%) | 2 (100%) | 1 | |
| 3 | Sugun et al (3). | 2010 | Turkey | Retrospective cohort study | Distal radius fractures | 46 | 59/230 screws (26%) | 13 (28.3%) | 6 | |
| 4 | Bianchi et al (4). | 2018 | United States | Retrospective case serie | Distal radius fractures | 9 | 1 (100%) | 1 (100%) | 4 | |
| 5 | Gyuricza et al (5). | 2011 | United States | Retrospective cohort study | Distal radius fractures | 28 | 28 (100%) | 28 (100%) | 15.75 | |
| 6 | Drobetz et al (6). | 2003 | Austria | Retrospective case series | Distal radial fractures | 49 | n/a | 6 (12.2%) | 26 | |
| 7 | Yamazaki et al (7). | 2009 | Japan | Case report | Open reduction and internal fixation for a displaced intraarticular fracture of the distal radius. | 1 | 1 (100%) | 1 (100%) | NA | |

| | | | | | | | | | | |
|---|---|---|---|---|---|---|---|---|---|---|
| 8 | Suk et al (8). | 2001 | Korea | Retrospective study | Thoracic pedicle screw fixation for spinal deformities | 462 | 48 (10.4%) | 4 (0.8%) | 24 | |
| 9 | Yang et al (9). | 2010 | China | Prospective clinical trial. | Treatment of proximal humerus fractures using a locked proximal humerus plate (LPHP). | 64 | 5 (7.6%) | 5 (7.6%) | 25 | |
| 10 | Oc et al (10). | 2018 | Turkey | Comparative study | Distal radius fractures | 47 | 12 (25.5) | 7 (14.9%) | N/A | |
| 11 | Rellan et al (11). | 2007-2015 | Argentina | Retrospective review | Treatment of distal radial fractures with volar locking plates | 992 | 9 (0.9%) | 9 (0.9%) | 12 | |
| 12 | Mayne et al (12). | 2009 | Canada | Case report | Total hip arthroplasty (THA) of the left hip. | 1/1 | 1 (100%) | 1 (100%) | 19 | |
| 13 | Alic et al (13). | 2023 | Turkey | Clinical study | Open reduction and volar plating for distal radius fractures | 29 | 23 (79.3%) | 15 (51.7%) | 16 | |
| 14 | Ahmed et al (14). | 2015 | United Kingdom | Retrospective study | Scaphoid fracture fixation | 56 | 9 (16%) | 9 (16%) | 5.25 | Pain |
| 15 | Garcia et al (15). | 2006 | Spain | Case report | Total hip arthroplasty | 1 | 1 (100%) | 1 (100%) | 6 | |
| 16 | Park et al (16). | 2021 | South Korea | Case report | Total hip arthroplasty | 1 | 1 (100%) | 1 (100%) | N/A | |
| 17 | Vargas-Reveron et al (17). | 2021 | Spain | Case report | Total hip arthroplasty | 1 | 1 (100%) | 1 (100%) | 36 | |
| 18 | Drosos et al (18). | 2006 | Greece | Case report | Distal tibial shaft fracture | 1 | 1 (100%) | 1 (100%) | 12 | |
| 19 | Zhu et al (19). | 2022 | China | Retrospective cohort study | Distal radius reoperation due to screw penetration | | 5 (1.7%) | 2 (40%) | 12 | |
| 20 | Esses et al (20). | 1993 | USA | Survey analysis. | Pedicle screw fixation in the lumbar spine. | 617 | - | 119 (19.3%) | | |

| | | | | | | | | | | |
|---|---|---|---|---|---|---|---|---|---|---|
| 21 | Hamill et al (21). | 2018 | Germany | Prospective cohort study | Pedicle of the lumbar vertebrae | 60 | 11 (18.3%) | 2 (3.3%) | 24 | Neurovascular |
| 22 | Starnoni et al (22). | 2019 | Italy | Case report | Scaphoid fracture | 1 | 1 (100%) | 1 (100%) | 2 | |
| 23 | Madawi et al (23). | 1997 | England and Hungary | Retrospective cohort study | C1–2 transarticular screw fixation. | 61 | 8 (13.1%) | 5 (8.2%) | 39 | |
| 24 | Lonstein et al (24). | 1999 | USA | Retrospective cohort study | Pedicle screw insertion into the spine (depending on diagnosis) | 875 | 134 (15.3%) | 68 (7.8%) | 36 | |
| 25 | Dias et al (25). | 2020 | UK | Retrospective cohort study | Surgical fixation of scaphoid fracture | 219 | 93 (65%) | 6 (4%) | 12 | |
| 26 | Hu et al (26). | 2013 | China & USA | Clinical study, radiographic assessment. | Posterior C-1 lateral mass screw fixation | 196 | 2 (1%) | 2 (1%) | 40.5 | |
| 27 | Kavuri et al (27). | 2018 | USA | Systematic review | Locking plate fixation for traumatic proximal humerus fractures. | 1342 | 128 (9.5%) | 22 (3.6%) | 12 | Non-union/Pseudoarthrosis |
| 28 | Chanbour et al (28). | 2020-2021 | USA | Retrospective cohort study. | Elective anterior cervical discectomy and fusion (ACDF). | 406 | 113 (27.8%) | 141 (34.7%) | 12 | |
| 29 | Muller et al (29). | 2000 | Germany | Retrospective cohort study | Anterior screw fixation of odontoid fractures | 28 | 3 (10.7%) | 1 (3.6%) | 15 | |
| 30 | Beeres et al (30). | 2017 | Switzerland | Multicentre retrospective case series. | Proximal Humerus reoperation rate for screw perforation | 282 | 65 (23%) | 127 (45%) | 12 | |
| 31 | Wang et al (31). | 2022 | China | Retrospective cohort study. | Internal fixation for femoral neck fractures in children. | 153 | - | - | 12 | Avascular Necrosis |

*Supplementary Table 1: Data Extraction Table demonstrating the extraction of various details used in the review.*

## BIBLIOGRAPHY


1. Bergsma M, Doornberg JN, Duit R, Saarig A, Worsley D, Jaarsma R, Lleyton Hewitt Study Group. Volar plating in distal radius fractures: A prospective clinical study on efficacy of dorsal tangential views to avoid screw penetration. Injury. 2018 Oct;49(10):1810–5.
2. Benson EC, DeCarvalho A, Mikola EA, Veitch JM, Moneim MS. Two potential causes of EPL rupture after distal radius volar plate fixation. Clin Orthop. 2006 Oct;451:218–22.
3. Sügün T, Karabay N, Gürbüz Y, Ozaksar K, Toros T, Kayalar M. Screw prominences related to palmar locking plating of distal radius. J Hand Surg Eur Vol. 2011 May;36:320–4.
4. Bianchi S, van Aaken J, Glauser T, Martinoli C, Beaulieu JY, Santa DD. Screw Impingement on the Extensor Tendons in Distal Radius Fractures Treated by Volar Plating: Sonographic Appearance. Am J Roentgenol. 2008 Nov;191(5):W199–203.
5. Gyuricza C, Carlson MG, Weiland AJ, Wolfe SW, Hotchkiss RN, Daluiski A. Removal of Locked Volar Plates After Distal Radius Fractures. J Hand Surg. 2011 Jun;36(6):982–5.
6. Drobetz H, Kutscha-Lissberg E. Osteosynthesis of distal radial fractures with a volar locking screw plate system. Int Orthop. 2003;27(1):1–6.
7. Yamazaki H, Hattori Y, Doi K. Delayed rupture of flexor tendons caused by protrusion of a screw head of a volar plate for distal radius fracture: a case report. Hand Surg. 2008 Jan;13(01):27–9.
8. Suk SI, Kim WJ, Lee SM, Kim JH, Chung ER. Thoracic Pedicle Screw Fixation in Spinal Deformities: Are They Really Safe? Spine. 2001 Sep;26(18):2049–57.
9. Yang H, Li Z, Zhou F, Wang D, Zhong B. A prospective clinical study of proximal humerus fractures treated with a locking proximal humerus plate. J Orthop Trauma. 2011 Jan;25(1):11–7.

10. Oc Y, Kilinc BE, Gulcu A, Varol A, Ertugrul R, Kara A. Ultrasonography or direct radiography? A comparison of two techniques to detect dorsal screw penetration after volar plate fixation. J Orthop Surg. 2018 Apr;13(1):70.

11. Rellán I, Gallucci GL, Boretto JG, Donndorff A, De Carli P. Secondary Tendinopathy After Distal Radius Volar Plate Fixation: Results of 8 Years' Experience. HAND. 2016 Sep;11(1_suppl):38S-38S.
12. Mayne IP, Kosashvili Y, White LM, Backstein D. Iliopsoas Tendonitis due to the Protrusion of an Acetabular Component Fixation Screw After Total Hip Arthroplasty. J Arthroplasty. 2010 Jun;25(4):659.e5-659.e8
13. Alic T, Fidan N, Hassa E, Zehir S. Is tendinitis in volar plating related to the dorsally protruding screw length and its compartment? Jt Dis Relat Surg. 2022 Dec;34(1):108–14.

14. Ahmed U, Malik S, David M, Simpson C, Tan S, Power D. The Headless Compression Screw - Technical challenges in scaphoid fracture fixation. J Orthop. 2015 Dec;12(Suppl 2):S211-216.

15. Garcia S, Popescu D, Gallart X, Riba J. Intrapelvic Protrusion of a Dynamic Hip Screw. HIP Int. 2006 Jan;16(4):301–4.

16. Lee NJ, Vulapalli M, Park P, Kim JS, Boddapati V, Mathew J, Amorosa L, Sardar Z, Lehman R, Riew K. Does screw length for primary two-level ACDF influence pseudarthrosis risk? Spine J. 2020 Nov;20(11):1752–60.
17. Vargas-Reverón C, Capurro B, Alías AJ, Muñoz-Mahamud E, Pifarré PT, Fernández-Valencia JA. Removal of protruding screws in a painful total hip arthroplasty: A case report. Radiol Case Rep. 2021 Jan;16(1):103–7.
18. Drosos GI, Stavropoulos NI, Kazakos KI. Peroneal nerve damage by oblique proximal locking screw in tibial fracture nailing: a new emerging complication? Arch Orthop Trauma Surg. 2007 Aug;127(6):449–51.
19. Zhu, Yanbin, Song Liu, Yansen Li, Qianru Yang, Ruoxiang Miao, and Yingze Zhang. 'Risk Factors for Complications Following Volar Locking Plate (VLP) Fixation of Unstable Distal Radius Fracture (DRF)'. Edited by Jun Zou. *BioMed Research International* 2022, no. 1 (January 2022): 9117533. https://doi.org/10.1155/2022/9117533.
20. Esses SI, Sachs BL, Dreyzin V. Complications Associated with the Technique of Pedicle Screw Fixation A Selected Survey of ABS Members: Spine. 1993 Nov;18(15):2231–9.
21. Hamill CL, Lenke LG, Bridwell KH, Chapman MP, Blanke K, Baldus C. The Use of Pedicle Screw Fixation to Improve Correction in the Lumbar Spine of Patients With Idiopathic Scoliosis: Is It Warranted? Spine. 1996 May;21(10):1241–9.
22. Starnoni M, Colzani G, De Santis G, Acciaro AL. Median Nerve Injury Caused by Screw Malpositioning in Percutaneous Scaphoid Fracture Fixation. Plast Reconstr Surg – Glob Open. 2019 Jun;7(6):e2292.
23. Madawi AA, Casey ATH, Solanki GA, Tuite G, Veres R, Crockard HA. Radiological and anatomical evaluation of the atlantoaxial transarticular screw fixation technique. J Neurosurg. 1997 Jun;86(6):961–8.
24. Lonstein JE, Denis F, Perra JH, Pinto MR, Smith MD, Winter RB. Complications Associated with Pedicle Screws*. JBJS. 1999 Nov;81(11):1519.
25. Dias JJ, Brealey SD, Fairhurst C, Amirfeyz R, Bhowal B, Blewitt N, Brewster M, Brown D, Choudhary S, Coapes C, Cook L, Costa M, Davis T, Di Mascio L, Giddins G, Hedley H, Hewitt C, Hinde S, Hobby J, Hodgson S, Jefferson L, Jeyapalan K, Johnston P, Jones J, Keding A, Leighton P, Logan A, Mason W, McAndrew A, McNab I, Muir L, Nicholl J, Northgraves M, Palmer J, Poulter R, Rahimtoola Z, Rangan A, Richards S, Richardson G, Stuart P, Taub N, Tavakkolizadeh A, Tew G, Thompson J, Togerson D, Warwick D.. Surgery versus cast immobilisation for adults with a bicortical fracture of the scaphoid waist (SWIFFT): a pragmatic, multicentre, open-label, randomised superiority trial. The Lancet. 2020 Aug;396(10248):390–401.

26. Hu Y, Kepler CK, Albert TJ, Yuan Z shan, Ma W hu, Gu Y jie, Xu R Ming. Accuracy and complications associated with the freehand C-1 lateral mass screw fixation technique: a radiographic and clinical assessment: Clinical article. J Neurosurg Spine. 2013 Apr;18(4):372–7.
27. Kavuri V, Bowden B, Kumar N, Cerynik D. Complications Associated with Locking Plate of Proximal Humerus Fractures. Indian J Orthop. 2018;52(2):108–16.

28. Chanbour H, Bendfeldt GA, Johnson GW, Peterson K, Ahluwalia R, Younus I, Longo M, Abtahi A, Stephens B, Zuckerman S. Longer Screws Decrease the Risk of Radiographic Pseudarthrosis Following Elective Anterior Cervical Discectomy and Fusion. Glob Spine J. 2023 Nov 11;21925682231214361.

29. Müller EJ, Wick M, Russe OJ, Palta M, Muhr G. Die direkte Verschraubung von Frakturen des Dens axis. Unfallchirurg. 2000 Jan 27;103(1):38–43.
30. Beeres FJP, Hallensleben NDL, Rhemrev SJ, Goslings JC, Oehme F, Meylaerts SAG, Babst R, Schep N.W.L. Plate fixation of the proximal humerus: an international multicentre comparative study of postoperative complications. Arch Orthop Trauma Surg. 2017 Dec;137(12):1685–92.
31. Wang W, Li Y, Xiong Z, Guo Y, Li M, Mei H, Shao J, Li J, Canavese F, Chen S. Effect of the Number, Size, and Location of Cannulated Screws on the Incidence of Avascular Necrosis of the Femoral Head in Pediatric Femoral Neck Fractures: A Review of 153 Cases. J Pediatr Orthop. 2022 Mar;42(3):149–57.
